# 

**Published:** 1892-07

**Authors:** 


**TITLE PAGE AND INDEX WITH THIS NUMBER.**

### CONTENTS FOR JULY, 1892.

ORIGINAL COMMUNICATIONS. PAGE.

An Overlooked Factor in the Production of Conjunctivitis. By JULIUS POHLMAN, M. D. 705
Six Cases of Abdominal Section. By M. A. CROCKETT, A. B., M. D. 710
The Bacteria of the Mouth—Of Interest to Dentists and Others. By FRANK J. THORNBURY, M. D. 715

SOCIETY PROCEEDINGS.

New York Academy of Medicine—Section on Orthopedic Surgery 718
Buffalo Academy of Medicine (Constitution and By-Laws) 728

PROGRESS IN MEDICAL SCIENCE.

Ophthalmology 736

EDITORIAL.

American Medical Association at Detroit 741
Topics of the Month 743

PERSONAL.

Dr. A. J. Martin—Dr. William Easterly Ashton—Dr. Robert Tuttle Morris 747
Dr. Lewis S. Pilcher—Dr. Arch Dixon—Dr. Alvin A. Hubbell—Dr. T. Haven Ross—Mr. William Dean Howells 748
Miss Jessamy Harte—Dr. Charles G. Stockton 749

SOCIETY MEETINGS.

American Neurological Association—The Medical Association of Central New York 749

OBITUARY.

Riley, Henry A., A. B., LL. B. 750

Book Reviews 750
Books Received 761

MISCELLANY.

The Law Governing the Practice of Medicine in New York State and How it is Enforced 762

